# Supplementary material for: Mental health among healthcare workers during COVID-19 pandemic in Thailand
Source: PLoS One. 2022 May 20;17(5):e0268704. doi: 10.1371/journal.pone.0268704 (PMC9122199; doi:10.1371/journal.pone.0268704)
Supplement: Attachment — Submitted filename: Response to reviewers.docx [file pone.0268704.s002.docx]

**Rebuttal letter**

___________________________________________________________

We thank the editor and the two reviewers for their comments on our manuscript. Below is

our response to each point raised by the academic editor and reviewers. We hope that we

satisfyingly addressed them and that the manuscript will be now suited for publication.

Sincerely,

On behalf of all authors,

Pantri Kirdchok

**Academic editor:**

1. Please ensure that your manuscript meets PLOS ONE's style requirements, including those for file naming. The PLOS ONE style templates can be found at

We have already ensured our manuscript meet the requirements.

2. Financial disclosure

We have changed the statement about funding to “This work was supported by Navamindradhiraj University Research Fund” on line 307.

3. Please provide additional details regarding participant consent

We have added details about participant consent **on lines 117-118.**

**Reviewer #1:**

**Q1: Under the Introduction, the authors stated " Since the World Health Organization declaration on March 2020 [1] the pandemic situation has been fluctuating, and the crisis has yet to be fully resolved". It is important to talk about the impact of pandemic on mental health based on the following landmark studies**

We cited the data from the recommended articles in the introduction part.

1. A systematic review of COVID-19 on mental health Impact of COVID-19 pandemic on mental health in the general population: A systematic review. J Affect Disord. 2020;277:55-64. **On line 56**
2. The impact of COVID-19 on three continents: A chain mediation model on COVID-19 symptoms and mental health outcomes in Americans, Asians and Europeans. SciRep 11, 6481 (2021). **On lines 56 and 60**
3. The impact of COVID-19 pandemic on physical and mental health of Asians: A study of seven middle-income countries in Asia. PLoS One. 2021 Feb 11;16(2):e0246824. **On line 56**
4. Government response moderates the mental health impact of COVID-19: A systematic review and meta-analysis of depression outcomes across countries. J Affect Disord. 2021 May 27;290:364-377. **On line 57**
5. Association Between Mood Disorders and Risk of COVID-19 Infection, Hospitalization, and Death: A Systematic Review and Meta-analysis. JAMA Psychiatry. 2021 Jul 28.  **On lines 58 and 61**

**Q2: Under the paragraph, "A number of studies also identified associated factors of negative mental health outcomes 63 among HCWs". Since Thailand belongs the Asia-Pacific, please comment on the research performed in this region based on the following landmark studies but not limited to the following studies Under the discussion, the authors should compare their findings with the findings from the following Asian countries:**

We cited the data from the recommended articles in the introduction and discussion part.

1. Perception Toward Exposure Risk of COVID-19 Among Health Workers in Vietnam: Status and Correlated Factors. Front Public Health. 2021 May 25;9:589317. **On lines 73**
2. The impact of COVID-19 pandemic on global mental health: From the general public to healthcare workers. Ann AcadMed Singap. 2021 Mar;50(3):198-199. **On lines 72, 94 and 241**
3. Asian-Pacific perspective on the psychological well-being of healthcare workers during the evolution of the COVID-19pandemic. BJPsych Open. 2020;6(6):e116. Published 2020 Oct 8. **On lines 79, 94 and 241**
4. A multinational, multicentre study on the psychological outcomes and associated physical symptoms amongst healthcare workers during COVID-19 outbreak [published online ahead of print, 2020 Apr 21]. Brain Behav Immun. 2020;S0889-1591(20)30523-7. **On lines 79, 94, 239, 241, and 261-262**
5. Psychological Impact of the COVID-19 Pandemic on Health Care Workers in Singapore [published online ahead of print,2020 Apr 6]. Ann Intern Med. 2020;M20-1083. **On lines 79, 94 and 241**
6. Impacts of COVID-19 on the Life and Work of Healthcare Workers During the Nationwide Partial Lockdown in Vietnam.Front Psychol. 2021 Aug 19;12:563193. doi: 10.3389/fpsyg.2021.563193. **On lines 79**

**Q3: Please discuss the impact on surgeons (beside phyisicians) during the pandemic based on the following study:**

We cited the data from the recommended articles in the discussion part.

1. Psychological Health of Surgeons in a Time of COVID-19: A Global Survey [published online ahead of print, 2021 Jan22]. Ann Surg. 2021;10.1097/SLA. 0000000000004775. **On lines 296-297**

**Q4: Under discussion, please mention online psychological treatment e.g. Internet Cognitive Behavior Therapy (iCBT) to help healthcare workers based on the following studies under "actionable measures should be taken into account to prevent and reduce mental health problems among healthcare worker"**

We cited the data from the recommended articles in the discussion part **on lines 285-287**.

1. Use of Cognitive Behavior Therapy (CBT) to treat psychiatric symptoms during COVID-19: Mental Health Strategies to Combat the Psychological Impact of COVID-19 Beyond Paranoia and Panic. Ann Acad MedSingapore. 2020;49(3):155‐160.
2. Cost-effectiveness of iCBT: Moodle: The cost effective solution for internet cognitive

behavioral therapy (I-CBT) interventions. Technol Health Care.2017;25(1):163-165.

3. Internet CBT can treat psychiatric symptoms such as insomnia: Efficacy of digital cognitive behavioural therapy for insomnia: a meta-analysis of randomised controlled trials. Sleep Med.2020 Aug 26;75:315-325.

**Q5: The authors found that "The issue was reported by 26 respondents (11.8%) who believed that having enough 228 personal protective equipment would improve their mental health." Under discussion, please discuss the findings of the following studies and how these studies support the above observation from psychoneuroimmunological and cultural perspectives:**

We cited the data from the recommended articles in the discussion part **on lines 279-280**.

1. Is Returning to Work during the COVID-19 Pandemic Stressful? A Study on Immediate Mental Health Status and Psychoneuroimmunity Prevention Measures of Chinese Workforce [published online ahead of print, 2020 Apr 23]. Brain Behav Immun. 2020;S0889-1591(20)30603-6.
2. The Association Between Physical and Mental Health and Face Mask Use During the COVID-19 Pandemic: A Comparison of Two Countries With Different Views and Practices. Front Psychiatry. 2020;11:569981. Published 2020 Sep9.

**Reviewer #2:**

**1. Abstract**

(1) Please use the full names when first time mention it

We correct the full names following the reviewer’s comment **on lines 25 and 44.**

(2) Methods: that would be great if the authors could add more information about the study methods, including the study setting, participants, data analysis methods and other information if needed.

We added the recommended details of methods **on lines 26-27, and 30-32.**

**2. Introduction**

(1) It would be useful to mention the background and context of the Thai healthcare system, what are the differences ofthe healthcare workers who working in Thailand compared with healthcare workers from other countries?

We cited the data from the recommended articles in the introduction part.

1. Thatrimontrichai, A., Weber, D. J., & Apisarnthanarak, A. (2021). Mental health among healthcare personnel during COVID-19 in Asia: A systematic review. Journal of the Formosan Medical Association. **On lines 68-70**
2. Marvaldi, M., Mallet, J., Dubertret, C., Moro, M. R., & Guessoum, S. B. (2021). Anxiety, depression, trauma-related, and sleep disorders among healthcare workers during the COVID-19 pandemic: a systematic review and meta-analysis. Neuroscience & Biobehavioral Reviews. **On lines 79**
3. Li, Yufei, Nathaniel Scherer, Lambert Felix, and Hannah Kuper. "Prevalence of depression, anxiety and post-traumatic stress disorder in health care workers during the COVID-19 pandemic: A systematic review and meta-analysis." PloS one16, no. 3 (2021): e0246454. **On lines 77**

(2) The authors should explain more healthcare workers’ mental health during COVID-19 outbreak, as the authors mentioned that “… psychological outcomes of healthcare workers (HCWs) during the pandemic have been extensively studied globally”.

We discussed the more about this issue **on lines 74-80.**

(3) Some sentences need provide reference(s), for example, “…..there was a study in Thailand finding that 42.5% of healthcare workers had anxiety”, by the way, please mention this study recruited participants during the COVID19 or not. All the information needs to focus on the topic of this study.

We added detailed the more about this issue **on line 96.**

**3. Methods**

(1) All the study methods related to qualitative study/methods are missing, the authors need to describe the qualitative study and the whole mixed methods design clearly.

Methodological orientation and theory are lacking. What was the reason that you chose a mixed-methods approach. It should be supported using literature. The type of mixed methods used in your study should be described.

(2) Research/study procedure is also missing. What are trained data collectors?

(1) and (2); We added more details following the reviewer’s comment **on lines 167-161.**

(3) Please provide the psychometric properties of the questionnaires used in this study.

We added the psychometric properties of the questionnaires **on lines 129-129, 134-134, and 139-140.**

(4) Since both qualitative and quantitative data analysis has been used, the heading should be changed to 'data analysis ‘instead of 'Statistical analysis'

We changed the word “Statistical analysis” to “Data analysis” **on line 149.**

**4. Results and Discussion**

(1) Data integration of quantitative and qualitative results is missing. The results section is unclear if the methods of this study is unclear. It is difficult to see how it is mixed with both quantitative and qualitative data.

No data integration of quantitative and qualitative were executed.

We modified the manuscript to address the points made by the editor and the reviewers. We agreed with the comments in all accounts. We believe that the manuscript is now more readable, more informative, and its conclusions more useful to the public.
