# Supplementary material for: Mental health among healthcare workers during COVID-19 pandemic in Thailand
Source: PLoS One. 2022 May 20;17(5):e0268704. doi: 10.1371/journal.pone.0268704 (PMC9122199; doi:10.1371/journal.pone.0268704)
Supplement: Attachment — Submitted filename: Response to reviewers_3.docx [file pone.0268704.s003.docx]

**Rebuttal letter**

___________________________________________________________

We thank the editor and the two reviewers for their comments on our manuscript. Below is

our response to each point raised by the academic editor and reviewers. We hope that we

satisfyingly addressed them and that the manuscript will be now suited for publication.

Sincerely,

On behalf of all authors,

Pantri Kirdchok

**Academic editor:**

1. Methods: please further elaborate the rationales for choosing mixed methods this study.

We choose a multiple method study design instead of mixed methods

2. Methods: For qualitative part, is there only one open-ended questions for qualitative data collection? any interview data? If only one open-ended question, content analysis might be quite more appropriate than the thematic analysis.

For the qualitative part, there are open-ended questions so, we use the content analysis instead of the thematic analysis method.

3. Methods/results: as you defined this study is a mixed methods study, but there is no any combination of the quantitative data and qualitative data. Combination is the key feature of mixed method study, otherwise it is much appropriate to define it as a multiple method study.

We define our study as a multiple method study, so there is no data integration of quantitative and qualitative were executed. In addition, we try to explain more about qualitative data in the method and discussion part.

**Reviewer #2:**

Thanks for the opportunity to review this manuscript again. I think the authors did not revise the manuscript with my comments appropriately, for example, the mixed-methods design in this study is still unclear and there are many methodological issues in this manuscript.

We define our study as a multiple method study using content analysis instead of thematic analysis because we asked only open-ended questions and there was no in-depth interview in this study. Furthermore, we try to explain more about qualitative data in the method and discussion part.

We modified the manuscript to address the points made by the editor and the reviewers. We agreed with the comments in all accounts. We believe that the manuscript is now more readable, more informative, and its conclusions more useful to the public.
